# MicroRNA Target Detection and Analysis for Genes Related to Breast Cancer Using MDLcompress

**DOI:** 10.1186/1687-4153-2007-43670

**Published:** 2007-10-30

**Authors:** Scott C Evans, Antonis Kourtidis, T Stephen Markham, Jonathan Miller, Douglas S Conklin, Andrew S Torres

**Affiliations:** 1 GE Global Research, One Research Circle, Niskayuna, NY 12309, USA; 2 Gen*NY*Sis Center for Excellence in Cancer Genomics, University at Albany, State University of New York, One Discovery Drive, Rensselaer, NY 12144, USA; 3 Human Genome Sequencing Center, Baylor College of Medicine, One Baylor Plaza, Houston, TX 77030, USA

## Abstract

We describe initial results of miRNA sequence analysis with the optimal symbol compression ratio (OSCR) algorithm and recast this grammar inference algorithm as an improved minimum description length (MDL) learning tool: *MDLcompress*. We apply this tool to explore the relationship between miRNAs, single nucleotide polymorphisms (SNPs), and breast cancer. Our new algorithm outperforms other grammar-based coding methods, such as DNA Sequitur, while retaining a two-part code that highlights biologically significant phrases. The deep recursion of *MDLcompress*, together with its explicit two-part coding, enables it to identify biologically meaningful sequence without needlessly restrictive priors. The ability to quantify cost in bits for phrases in the MDL model allows prediction of regions where SNPs may have the most impact on biological activity. *MDLcompress* improves on our previous algorithm in execution time through an innovative data structure, and in specificity of motif detection (compression) through improved heuristics. An *MDLcompress* analysis of 144 over expressed genes from the breast cancer cell line BT474 has identified novel motifs, including potential microRNA (miRNA) binding sites that are candidates for experimental validation.

## 

[1,2,3,4,5,6,7,8,9,10,11,12,13,14,15,16,17,18,19,20,21,22,23,24,25,26,27,28,29,30,31,32,33,34,35,36,37,38,39,40]
